# The global spread of Middle East respiratory syndrome: an analysis fusing traditional epidemiological tracing and molecular phylodynamics

**DOI:** 10.1186/s41256-016-0014-7

**Published:** 2016-09-28

**Authors:** Jae Min, Eleonora Cella, Massimo Ciccozzi, Antonello Pelosi, Marco Salemi, Mattia Prosperi

**Affiliations:** 1 Department of Epidemiology, College of Public Health & Health Professions and College of Medicine, University of Florida, 2004 Mowry Rd, Gainesville, FL 32610-0231 USA; 2 Department of Infectious, Parasitic and Immune-mediated Diseases, National Institute of Health, Viale Regina Elena, 299, 00161 Rome, Italy; 3 Department of Public Health and Infectious Diseases, Sapienza University of Rome, Piazzale Aldo Moro, 5, 00185 Rome, Italy; 4 Department of Pathology, Immunology and Laboratory Medicine, Emerging Pathogens Institute, University of Florida, 2055 Mowry Rd, Gainesville, FL 32611 USA; 5 Department of Clinical Pathology and Microbiology Laboratory, University of Biomedical Campus, Via Alvaro del Portillo, 21, Rome, Italy

**Keywords:** Middle East Respiratory Syndrome, Coronavirus, Epidemiology, Phylodynamics

## Abstract

**Background:**

Since its discovery in 2012, over 1700 confirmed cases of Middle East Respiratory Syndrome (MERS) have been documented worldwide and more than a third of those cases have died. While the greatest number of cases has occurred in Saudi Arabia, the recent export of MERS-coronavirus (MERS-CoV) to Republic of Korea showed that a pandemic is a possibility that cannot be ignored. Due to the deficit of knowledge in transmission methodology, targeted treatment and possible vaccines, understanding this virus should be a priority. Our aim was to combine epidemiological data from literature with genetic information from viruses sequenced around the world to present a phylodynamic picture of MERS spread molecular level to global scale.

**Methods:**

We performed a qualitative meta-analysis of all laboratory confirmed cases worldwide to date based on literature, with emphasis on international transmission and healthcare associated infections. In parallel, we used publicly available MERS-CoV genomes from GenBank to create a phylogeographic tree, detailing geospatial timeline of viral evolution.

**Results:**

Several healthcare associated outbreaks starting with the retrospectively identified hospital outbreak in Jordan to the most recent outbreak in Riyadh, Saudi Arabia have occurred. MERS has also crossed many oceans, entering multiple nations in eight waves between 2012 and 2015. In this paper, the spatiotemporal history of MERS cases, as documented epidemiologically, was examined by Bayesian phylogenetic analysis. Distribution of sequences into geographic clusters and interleaving of MERS-CoV sequences from camels among those isolated from humans indicated that multiple zoonotic introductions occurred in endemic nations. We also report a summary of basic reproduction numbers for MERS-CoV in humans and camels.

**Conclusion:**

Together, these analyses can help us identify factors associated with viral evolution and spread as well as establish efficacy of infection control measures. The results are especially pertinent to countries without current MERS-CoV endemic, since their unfamiliarity makes them particularly susceptible to uncontrollable spread of a virus that may be imported by travelers.

**Electronic supplementary material:**

The online version of this article (doi:10.1186/s41256-016-0014-7) contains supplementary material, which is available to authorized users.

## Background

**Fig. 1:**
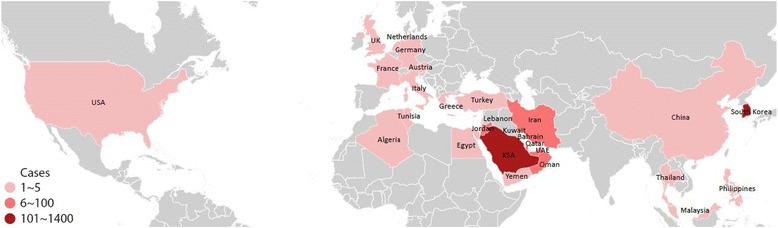
Map of MERS cases reported worldwide as of June 2016 according to WHO [1]. Countries in grey have had no cases of MERS. Light pink color indicates countries that reported less than or equal to 5 cases, including imported cases (Algeria, Austria, Bahrain, China, Egypt, France, Germany, Greece, Italy, Kuwait, Lebanon, Malaysia, Netherlands, Philippines, Thailand, Tunisia, Turkey, UK, United States, and Yemen), medium pink color shows countries with greater than 5 but less than 100 cases (Iran, Jordan, Oman, Qatar, and United Arab Emirates), and red colored countries have had more than 101 cases (Saudi Arabia and Korea)

**Fig. 2:**
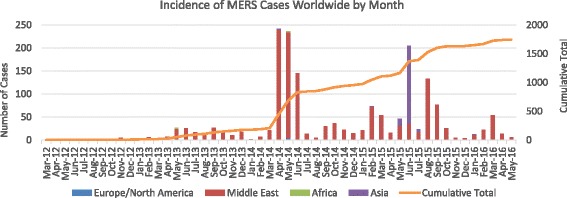
Summary of MERS cases from March 2012 to May 2016. Incidences summarized by month and by 4 regions of the globe where infected patients have been found: Middle East, Europe/North America, Africa, and Asia. Orange line indicates cumulative number of cases

After the identification of a novel coronavirus (CoV) in 2012, later named Middle East Respiratory Syndrome (MERS)-CoV, there has been a rise in cases reported worldwide. As of June 23, 2016, the World Health Organization (WHO) reported 1768 confirmed cases and 630 deaths, yielding case fatality of 35.6% [1]. Cases have been reported from 27 countries, spanning European, Asian, North American and African continents (Fig. 1). Majority of the cases occur in the MERS endemic region of Middle East, but the recent export of the virus to Korea showed that MERS-CoV can spread rapidly if unchecked by early detection and infection control measures [2]. The number of cases has climbed since its initial discovery, with just 9 cases identified in 2012, 168 in 2013, 768 in 2014, 683 in 2015 and 140 so far in 2016 (Fig. 2).

### MERS-CoV

MERS-CoV is the second coronavirus after Severe Acute Respiratory Syndrome (SARS)-CoV with the potential to cause a pandemic [3]. Characterized by its ‘corona’ or crown shape, it is a single-stranded RNA virus with approximately 30,000 nucleotides in its genome.

While current literature mostly concur that camels are important zoonotic reservoirs for MERS-CoV, there has been some evidence that bats might be primary viral reservoirs and that MERS-CoV will jump to a different host such as dromedary camels, and subsequently humans, when opportunities arise; similar transmission dynamic has been observed with SARS-CoV where palm civets acted as intermediary host between bats and humans [4, 5]. However coronaviruses isolated from bats are more genetically distant from human MERS-CoV than those isolated from camels, which have shown very high similarities to humans’ [6].

Virus transmission from camels is thought to be connected to consuming camel milk or urine, working with camels and/or handling camel products [7]. Secondary transmissions are largely associated with hospitals or close contacts of MERS cases. As shown with the family cluster in the United Kingdom (UK), relatives or people living in close contact with an infected patient are susceptible to the pathogen even outside of hospital settings [8].

While the exact methods of MERS-CoV transmissions are unknown respiratory droplet and aerosol transmissions are cited as most probable, but there is no conclusive evidence on how close a person has to be for exposure and what protection is most suitable [3, 9]. For example, the Korean Ministry of Health and Welfare (KMoH) classified close contacts as those who were within 2 meters of MERS-CoV infected patient or in contact with respiratory droplets without personal protective equipment, yet the extent to which of those close contacts were infected is unknown [10]. It has been hypothesized that camels can transmit a higher dose of viruses to humans while the quantity is lower between humans, and that MERS-CoV has not fully adapted for human-to-human transmission [11, 12]. Food, oral-fecal, and fomite transmissions are also possible transmission routes since the virus has been detected in camel milk, patient fecal sample and hospital surfaces [13–16].

### Middle East Respiratory Syndrome

Patients with MERS have a wide range of symptoms from being completely asymptomatic to suffering from severe respiratory illnesses. Fever cough, chills and myalgia are some of the most commonly reported symptoms in mild cases, but respiratory distress, kidney failure and septic shock have been reported in acute cases [17, 18]. There are neither vaccines nor specific medications against MERS-CoV, so treatments are usually palliative in nature [17, 19]. More than a third of those infected with MERS-CoV die [1]. For comparative perspective, case fatality was one in ten for the SARS pandemic of 2003 [20].

Research is yet to be done on the relationship between symptoms and transmissibility. Given that clinical procedures for acute patients can generate aerosolized viral particles, patients with severe respiratory distress would be more likely to transmit the virus compared to asymptomatic patients, but transmissibility of airborne MERS-CoV is unknown [12]. In addition, research in transmission is essential with regards to ‘superspreaders’ who are sources for large number of cases for healthcare associated outbreaks [21].

### Possibility of global outbreak

The uncertainty of pathogen transmission, lack of vaccine against MERS-CoV and deficit in MERS specific treatments make public health interventions challenging to design [22]. With ease of international travel, the possibility of MERS spread is present in all nations. Notably, countries without MERS endemic are unfamiliar with the infectious agent that may be imported by travelers and are, therefore, particularly vulnerable.

In this paper we reviewed epidemiological contact tracing information from public health agencies and peer-reviewed literature, in order to see geographic and temporal distribution of MERS cases around the globe. Concurrently, we used genetic sequences to infer transmission dynamics and inter-host evolution of MERS-CoV. The combined analysis was used to present a phylodynamic picture, detailing international, zoonotic and healthcare associated transmissions at genetic and population levels. These analyses can be used to understand pathogen spread and to implement public health measures to curb a pandemic [23].

## Methods

### Literature review

Detailed review of current literature was conducted using the five-step model of Khan et al. [24]. The literature searches on PubMed used following keywords: “Middle East Respiratory Syndrome”, “MERS” and “MERS CoV”. The article’s potential relevance to our topic was examined initially by article title then by abstract content. Included papers were case reports and articles on phylogenetics, healthcare related outbreaks and epidemiology, and we excluded papers on viral structure and model organism research. Relevant publications from national and international public health agencies were reviewed as well. WHO’s Disease Outbreak News and MERS risk assessments spanning September 2012 to June 2016 were used as primary sources to create a map of global transmissions [1]. The data were supplemented with the European Centre for Disease Prevention and Control (ECDC)’s Communicable Disease Threats Reports and Korean Center for Disease Control and Prevention (KCDC) and KMoH reports published online [25, 26]. Number of cases in hospital settings and non-hospital settings were compared using Mann-Whitney U test in SAS software v9.4 for Windows [27]. Following the literature review, forest plots of basic reproduction numbers were created using DistillerSR Forest Plot Generator [28].

### MERS-CoV genetic sequences

MERS-CoV sequences isolated from humans and camels were downloaded from the GenBank repository and are listed in Additional file 1 [29]. Combined Open Reading Frames 1a and 1b (ORF1a/b) region was chosen specifically for the high number of sequences available and low phylogenetic noise in order to create the most informative phylodynamic analyses. Duplicate sequences from single patients were excluded to explore inter-host dynamics without intra-host evolution, and only sequences with known date and place (city, state or country) of collection were included. Based on the metadata associated with the GenBank accession, we created three datasets: first with all CoV sequences isolated from both humans and camels, second dataset with solely sequences from humans, and third only with sequence from camels.

The sequences were aligned using Clustal X and manually edited using BioEdit [30, 31]. Then the best fitting nucleotide substitution model was chosen via hierarchical likelihood ratio test with ModelTest implemented in PAUP*4.0 [32, 33].

### Preliminary analysis

Three preliminary analyses were performed prior to Bayesian analysis: recombination, phylogenetic signal and temporal signal tests. First, identification of recombinant strains was done using Bootscan/Recscan method in RDP4 with default window size and parameters [34, 35].

Second phylogenetic signals in the datasets were investigated with TREE-PUZZLE [36]. It reported maximum likelihood for each new tree generated as a single dot on a triangle. The distribution of these dots among the seven zones of the triangle indicated their phylogenetic signals: if a dot was located in the central triangle, its tree had unresolved phylogeny with star-like structure. If a dot was located in the sides of the triangle, the phylogeny was network like and only partially resolved. Lastly, if the dot was positioned in one of the three corners, the tree topology was considered fully resolved [37].

Third, amount of evolutionary change over time, or temporal signal, from the sequences was examined using TempEst [38]. The three datasets were analyzed separately using default parameters.

### Coalescent model for demographic history

In order to conduct Bayesian phylogenetic analyses on MERS-CoV evolution three parameters for demographic growth model were tested: (1) molecular clock, (2) prior probability distribution and (3) marginal likelihood estimator. First, molecular clock was calibrated using dates of sample collection with the Bayesian Markov Chain Monte Carlo (MCMC) method in BEAST v1.8 [39]. Both strict and relaxed molecular clocks were tested, but relaxed clock with underlying lognormal distribution was superior. For the investigation of demographic growth of MERS-CoV ORF1a/b, four independent MCMC runs were carried out each with one of the following coalescent prior probability distributions: constant population size, exponential growth, Bayesian Gaussian Markov Random Field (GMRF) skyride plot and Bayesian skyline plot [40–42]. Both parameters listed above were tested with path sampling and stepping stone marginal likelihood estimators [43, 44].

Best fitting model was chosen by comparing the Bayes Factors [45]. If log (Bayes Factor) was >1 and <3, there was weak evidence against the model with lower marginal likelihood [43]. Higher log values indicated stronger evidence, such that values greater than 3 and 5 were considered to give strong and very strong evidence, respectively.

For all three datasets, the MCMC sampler was run for at least 50 million generations, sampling every 5000 generations. Only parameter estimates with effective sample size (ESS) greater than 250 were accepted to ensure proper mixing of the MCMC.

### Phylogeographic analyses

Phylogeographic analyses were conducted also using Bayesian-MCMC method in BEAST using (1) best fitting nucleotide substitution model chosen by ModelTest, (2) relaxed molecular clock with underlying lognormal rate distribution, (3) GMRF skyride plot as demographic model and (4) evolutionary rate previously estimated with sample collection dates. For all three datasets, the MCMC chains were run for at least 100 million generations and sampled every 10,000 steps. Using the standard continuous time Markov chain over discrete sampling locations (31 cities and countries) and Bayesian Stochastic Search Variable Selection procedures to infer social network, phylogeographic analyses were carried out for total and human datasets [46]. For the camel subset, continuous geographical trait analysis was used since spatial distribution of the MERS-CoV from camels was presumed not to be independent of their genetic phylogeny [47].

Maximum clade credibility trees which had the largest product of posterior clade probabilities, were selected after 10% burn-in using Tree Annotator, part of the BEAST package [39]. Calculations of posterior probability were used to establish statistical support for monophyletic clades.

## Results

### Phylodynamic analysis

We retrieved 196 MERS-CoV sequences from GenBank 88 from camels and 108 from humans, to analyze molecular evolution of the coronavirus over time (Additional file 1). All available ORF1a/b sequences as of March 16, 2016 were included and their dates ranged from April 2012 to the latest deposited sequences of June 2015. These sequences were from: China, Egypt, France, Germany, Oman, Qatar, Saudi Arabia, Korea, Thailand, UAE, UK and US [48]. Main dataset with all sequences, subset with only sequences from humans, and third subset with sequences from camels were created.

Sequences were aligned with Clustal X and four were removed due to poor alignment (GenBank accession numbers: KJ361499 KJ156916, KJ156941 and KJ156942).

Due to possibilities of recombinant MERS-CoV strains, aligned sequences were tested for recombination via RDP4 [49–51]. Sequences 441SA15 and 451SA15 had similarities to two different strains, 470SA15 and 355SA13 and were removed from the dataset since recombinant strains could convolute the evolutionary analysis (results shown in Additional file 2: Figure S1).

Then the phylogenetic content of ORF1a/b region was investigated by likelihood mapping method to ensure that there were enough signals to compute phylogenies [36]. The percentage of dots, or noise level, falling in the central triangle was 1.4% for all sequences, 2.3% for human sequences only, and 3.2% for camel sequences only (Additional file 2: Figure S2a, b and c respectively). For all three datasets, the three corners of the triangles summed to be greater than 95% and the central triangles had less than 30% noise, so we expected fully resolved, tree-like phylogenies [37].

Temporal signal of the sequences was also investigated. Temporal signal refers to how much genetic change has taken place between sampling times which is especially important in this case since samples were collected over time [38]. In order to make molecular clock inferences, genetic distances on phylogenetic trees have to be translated into temporal distances. Root-to-tip divergence plots are shown in Additional file 2: Figure S3a, b and c; no major deviations from the regression line were observed indicating that the genetic divergence of the sequence more or less align with what is expected given the sequence sampling date. The regression plots for the main dataset and the human subset had R^2^ values of 0.75 and 0.63, respectively, indicating a strong association while the camel subset had R^2^ of 0.42 which was weaker but still positive.

**Fig. 3:**
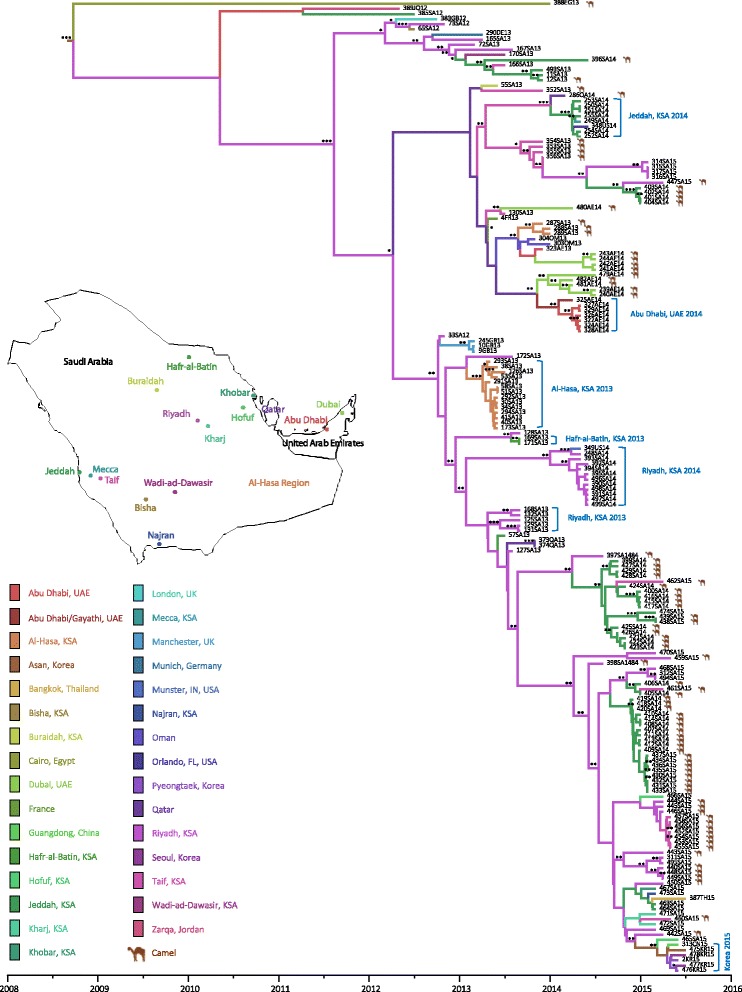
Time-scaled phylogeographic tree of MERS-CoV ORF1a/b sequences isolated from humans and camels. Each color shown in legend represents city or region of sampled sequence (tip branches) as well as ancestral lineage (internal branches) inferred by Bayesian phylogeography. Vertical blue lines encompass sequences collected at the time of outbreaks and brown camel symbols indicate sequences isolated from camels. An asterisk (*) along the branch represents the posterior probability for each clade greater than 0.90. Double asterisks (**) indicate > 0.95. Triple asterisks (***) indicate >0.99

**Fig. 4:**
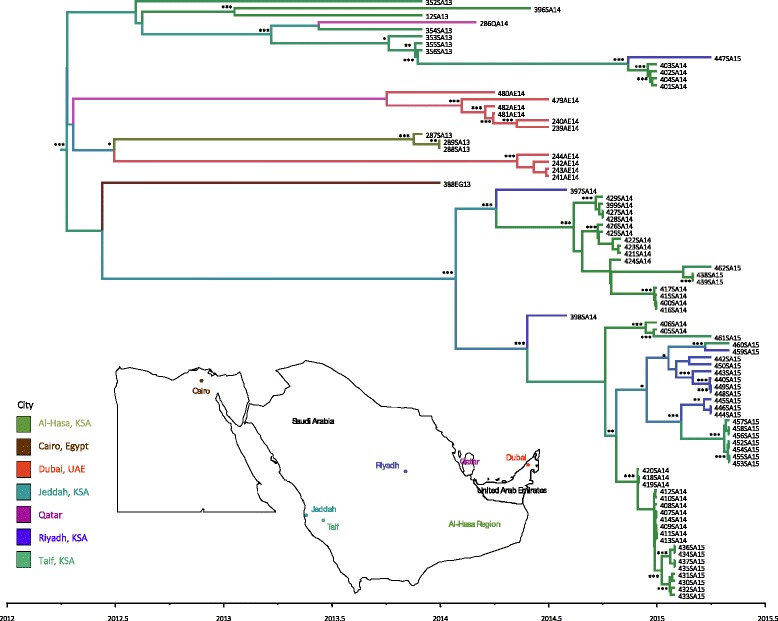
Time-scaled phylogeographic tree of MERS-CoV ORF1a/b sequences isolated from camels. Each color shown in legend represents city or region of sampled sequence (tip branches) as well as ancestral lineage (internal branches) inferred by Bayesian phylogeography. * represents posterior probability for the clade >0.90. ** for >0.95 and *** for >0.99

Finally the datasets were analyzed for their evolutionary history over time and space using BEAST. The Tamura-Nei 93 (TN93) evolutionary model with gamma distribution G = 0.05 was chosen by ModelTest as best fit. The GMRF skyride plot with stepping stone marginal likelihood estimator was selected as the best demographic model by BEAST. Phylogeographic trees of the main dataset is shown in Fig. 3 and the camel set is shown in Fig. 4 (main dataset colored according to countries is available as Additional file 2: Figure S4 and human set trees are included as Additional file 2: Figures S5 and S6).

### Overview of outbreaks and exports

**Fig. 5:**
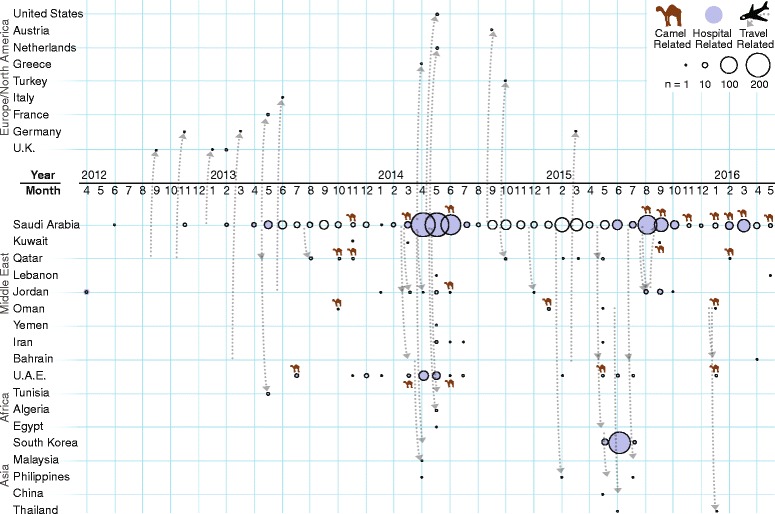
Worldwide epidemiological contact tracing summarized for MERS cases from April 2012 to May 2016. Countries are listed on the left and timeline is shown across the middle as x-axis, years indicated on top of the horizontal black line and months below. Circles represent the number of MERS cases reported for the month and blue color is used to indicate times when hospital related clusters were reported. Brown camel symbols indicate possible zoonotic transmissions due to handling of camels or consumption of its products. Key is shown in the upper right hand corner. Arrows indicate travel of MERS cases, with the tail of the arrow indicating country the patient visited prior to getting diagnosed in the country indicated by the arrowhead. In case of multinational travel, extra arrowheads are used to represent travel stops

Over 1300 MERS cases, or almost 80% of global incidence, have been reported from Saudi Arabia [1]. Korea has the second highest number of cases (n = 185) and United Arab Emirates (UAE) with 83 cases is third [1]. In the past three years since discovery of MERS-CoV, eight major healthcare associated outbreaks have been reported worldwide; the ninth wave recently occurring in multiple cities of Saudi Arabia. Those described here are based on cases with documented nosocomial transmission and are indicated by blue colored clusters in Fig. 5. Cases of MERS patients traveling internationally are indicated by arrows representing the direction of their travel and probable zoonotic transmission cases are marked by brown camels in the same figure.

The first hospital related outbreak was in Zarqa, Jordan where 2 cases were confirmed for MERS-CoV and 11 were declared probable cases retrospectively [52]. At the time of this outbreak in April, source of the respiratory illnesses was unknown, but it was later determined to be identical to the novel coronavirus (MERS-CoV) identified in the following September [52–54]. In total, nine cases were reported in 2012, and these early sequences, 389JO12, can be seen at the top of the tree in Fig. 3.

In January of 2013, UK reported an intra-familial cluster of 3 where the index case had traveled to Pakistan and Saudi Arabia [8]. The three cases (245GB13, 9GB13 and 10GB13 in Fig. 3) formed a distinct cluster in our tree, as expected, since the viruses isolated would have great genetic similarity consistent with proximity of the dates and location of infection. Secondary transmission occurred in France where the CoV was transmitted to a patient sharing a hospital room with a MERS confirmed case who had traveled to UAE [55, 56].

A major outbreak at several hospitals in the Al-Hasa region of Saudi Arabia occurred around April 2013 where epidemiological investigation linked 23 MERS cases to dialysis and intensive care units [57–59]. These are indicated in orange near the middle of the phylogenetic tree (Fig. 3). From June to August of the same year, there was a community outbreak in Hafr al-Batin where 12 patients were infected [60]. Although there was a transmission to a healthcare worker, the rest of the patients in this cluster were presumed to have gathered for the large, regional camel market, and therefore, both zoonotic and human-to-human transmissions may have occurred simultaneously. Interestingly, both the Al-Hasa and Hafr-al-Batin outbreaks cluster with sequences from Riyadh, which may indicate travel of persons, and possibly MERS-CoV with them, to and from this capital city.

In the following year from March to June of 2014, the biggest outbreak to date was reported from multiple countries: Saudi Arabia, UAE, Iran and Jordan [61–64]. Sequences from patients during this time distinctly separated into three clusters, Jeddah cluster (in green), Abu Dhabi cluster (red) and Riyadh cluster (pink), and they can be seen forming independent clades on the tree. And concurrently, there were at least 13 cases of exported MERS in UAE, Jordan, Philippines, Malaysia, Algeria, Egypt, Greece, Netherlands, Qatar, United States (US), Turkey and Austria via travelers who visited Saudi Arabia as shown in Fig. 5 [1, 26, 65–67].

In 2015, a traveler who visited the Arabian Peninsula was the index case in the largest outbreak of MERS outside of the Middle East, affecting 185 patients and inciting national panic in South Korea [2, 19, 68]. Nosocomial transmissions were reported from six hospitals, especially prevalent in two major medical centers, where 3 superspreaders have been linked to 166 cases [2]. When total number of cases are compared, nosocomial incidence is distinctly greater than incidence of community acquired or zoonotic infections (size differences are statistically significant, p < 0.0001) and superspreading may be the culprit. Son of a Korean MERS patient traveled to China and tested positive there (313CN15) [69]. The cluster involving Korean and Chinese samples seem most closely related to Saudi Arabian strain (465SA15) which was isolated from Hofuf.

In the Middle East, there was an outbreak in King Abulaziz Medical Center in Riyadh with 130 cases from June to August as well as multi-facility outbreaks in Riyadh and Madinah from September to November of 2015 [1, 26, 70]. In 2016, an outbreak in Buraidah of Saudi Arabia occurred in February and March [1, 26]. And recently, an outbreak in a Riyadh hospital with 24 cases due to superspreading occurred mid-2016 [71].

### Basic reproduction number

**Fig. 6:**
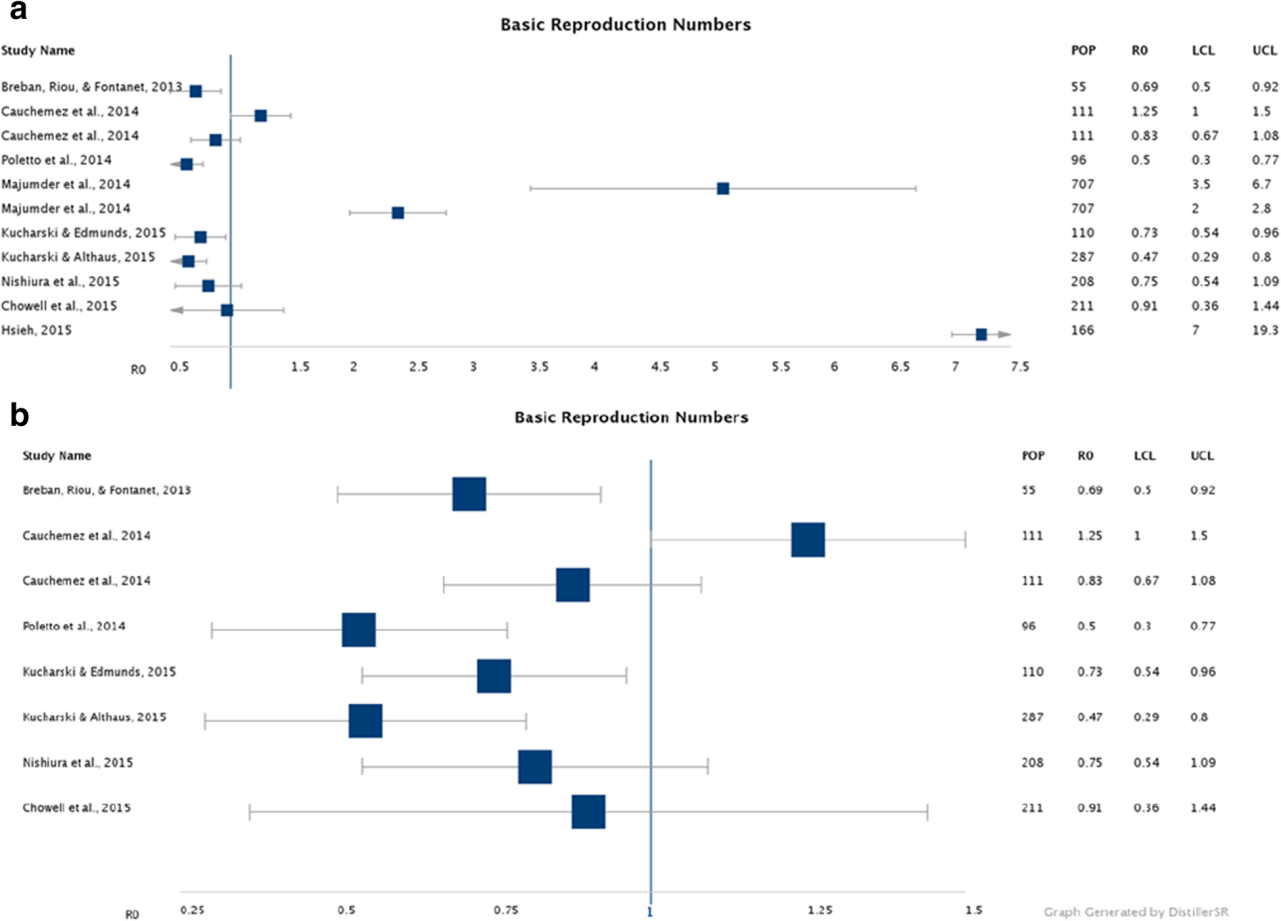
Summary of reported basic reproduction numbers from literature. (**a**) includes all basic reproductive numbers found through literature search. (**b**) Close-up excludes hospital specific outbreak R_0_’s

A wide range of basic reproduction numbers (R_0_) from 0.32 to 1.3, has been reported for MERS and are summarized in Fig. 6 [9, 14, 21, 72–79]. Most researchers agree that MERS has a low potential to become a pandemic at this point in time, but once the CoV is introduced in a nosocomial setting, the R_0_ can range from 2 to 6.7 and even from 7 to 19.3 [75, 78].

### Zoonotic transmissions

After two farm workers tested positive for the CoV, Qatar and WHO carried out an investigation of camel related MERS cases in October 2013 [80]. All 14 of their camels tested positive for MERS-CoV antibodies and 11 were positive for the virus itself. The partial sequences of the MERS-CoV sampled from camels were found genetically similar to the human samples. Parallel case was observed in Saudi Arabia, where researchers were able to pinpoint the specific camel that carried a 100% genetically identical virus as the patient [81]. These sequences (11SA13 and 12SA13) can be seen clustering together at the top of Fig. 4.

Sequences from camels can be seen interspersed throughout the tree in Fig. 3. When the camel sequences are examined alone (Fig. 4) it is easy to see that MERS-CoV from UAE form distinct clusters on their own (in red) while those from Saudi Arabia seem to have multiple clades. In our camel subset analysis, the United Arab Emirates clade diverged from Saudi Arabian sequences early, right after our estimated time to most recent common ancestor (tMRCA) of all taxa (March 2012 with 95% confidence intervals: July 2011-November 2012). This clade also includes some Al-Hasa sequences; Al-Hasa is the easternmost region of Saudi Arabia and therefore, geographically closest to UAE. The intermixing of Jeddah and Riyadh sequences in the bottom half of the tree may be explained by the camel trades that occur between these two large cities.

We analyzed the data separately for sequences isolated from humans and camels in order to ascertain when the introduction of MERS-CoV occurred in humans. For the main dataset with sequences from humans and camels, tMRCA was estimated to be September 2008 (95% Confidence Interval: September 2005-June 2011) of Riyadh, Saudi Arabia origin. For the human subset, tMRCA for all taxa was March 2011 (CI: June 2009-November 2011), and for the camel subset, tMRCA was March 2012 (CI: July 2011-November 2012) as mentioned above.

## Discussion

There were three aims for this paper: (1) to examine case incidence trends over time and geographic area using epidemiological information, (2) to trace evolutionary history of the virus in circulation using genetic data and (3) to explore ways in which these two analyses can be combined to design public health interventions.

While we aimed to have a thorough and complete representation of all contact tracing conducted the data may not be exhaustive. We chose PubMed as the source for peer-reviewed literature since it contains relevant articles from life science and medical journals, and we believe the flexibility in article search and use of MeSH terms are its strong points. In addition, the database and GenBank are overseen by the same organization (National Library of Medicine), which allowed us to link sequence meta-data with literature. However, PubMed does not include dissertations and conference proceedings that may be relevant and may be biased against articles written in languages other than English.

Non-heterogeneous sequencing of MERS-CoV is a limitation of this study. Some patients have been sampled and had their MERS-CoV sequenced multiple times while others were not reached. Duplicate sequences from single patients were excluded based on available meta-data since sampling bias can create apparent sinks that are not present in reality [82]. Better sampling and meta-data annotation would greatly aid further analysis in this regard.

Saudi Arabian sequences dominate the phylogeographic tree with greatest number of sequences and large number of probable ancestors (Figs. 3 and 4). This was expected since overwhelming majority of MERS cases have been reported there. Although Saudi Arabia had the largest amount of genetic sequences available which may skew the phylogenetic analysis, it also had the most number of MERS cases. Thus, the number of sequences was relatively proportional to the number of cases for the country. Saudi Arabia was the most frequent ancestor for many foreign exports, and phylogenetic data indicated that the direction of probable transmission was always from MERS endemic region of Middle East to non-endemic regions, such as Europe and Asia.

It has previously been posited that an area with great population such as central Saudi Arabia, may be a hub of genetically diverse MERS-CoV’s, introduced by passage of people and animals [83]. For example, the two cases exported to US were genetically dissimilar even though they were both healthcare workers returning from Saudi Arabia, the 348US14 sequence clustered with the hospital outbreak of Jeddah 2014 while the other US sequence 349US14 formed a distinct clade with Riyadh [84]. The Jeddah sequences seem most genetically similar to MERS-CoV isolated from a camel in Qatar (286QA14), even though Jeddah is located in the West coast of Saudi Arabia and Qatar borders the East. The intermixing of geographic backgrounds and phylogenetic clustering seem consistent with the theory on presence of several circulating MERS-CoV’s in Arabian Peninsula due to movement of animals and people. Geographical distribution of camels and human MERS-CoV cases are found to be highly correlated [85]. Since majority of human cases have been concentrated in the Middle East and camels in this region showed high seroprevalence of MERS-CoV antibodies, there has been ongoing testing in other camel dwelling regions, such as Africa and Asia [86–89]. Seropositivity ranging from 14.3 to 100% have been found in countries with dromedaries and a nice summary covering these studies is provided by Omrani et al. [90].

The later tMRCA for the camels (March 2012) relative to tMRCA for CoV isolated from humans (March 2011), was unexpected since antibodies against MERS-CoV have been detected in camels from samples archived as early as 1980s, but this may be due to dearth of early sequences from camels [87, 88, 91]. Earliest CoV isolates from camels were sequenced in 2013 and the short sampling timeframe from 2013 to 2015 makes the root of the tree less than reliable [92, 93]. While antibodies have been isolated from archived samples, the virus itself has not been successfully recovered; these historical samples may have been isolated in the latter part of the infection or much later after virus has cleared from the system. Other studies have shown that the virus most likely circulated in camels first becoming genetically divergent even within a single country, before jumping to human hosts, and because of the wide prevalence of seropositivity along with different lineages of CoV in camels, more MERS-CoV infections from camels to humans are bound to occur in the future [49, 87]. Nevertheless, this hypothesis cannot be validated without testing for MERS-CoV antibodies or even MERS-CoV in historical human samples [17].

Seasonality of MERS has been previously hypothesized from the high incidence of cases during spring and early summer months and it has been postulated that this may be correlated to the camel birthing pattern; young, newborn camels encountering MERS-CoV for the first time may get ill and transmit the virus to humans [13, 94, 95]. However, outbreaks in latter half of 2015 and early 2016 countered against the expected seasonality [96].

Infectiousness of MERS-CoV isolated from camels has been demonstrated by Raj et al., and shepherds and slaughterhouse workers have been shown to have 15 to 23 times higher proportion of individuals with MERS antibodies than the general population [97, 98]. And zoonotic transmission is an important factor to take into account since evolutionary rates of the virus may be different in humans and camels [99]. As MERS-CoV in camels seem to be much more prevalent and as shown here, genetically diverse, contacts with camels should be engaged with proper personal protection equipment, and public awareness about MERS-CoV infection related to camels should be raised.

In surveys conducted regarding MERS awareness of the Saudi Arabian public, less than half (47.1% and 48.9%) were aware that bats and camels could be primary sources of MERS-CoV [100, 101]. And there was an optimistic outlook on the fatality rate and treatment of the viral infection which may in turn be factors keeping the mild cases from seeking medical attention. Only half of the pilgrims surveyed by Alqahtani et al. were aware of MERS and about quarter stated drinking camel milk or visiting a camel farm as possible activities to be pursued during Hajj [102]. In a 2013 study, no MERS-CoV was detected amongst over 1000 pilgrims tested, but 2% of those tested in 2014 were positive [103, 104]. The crowded living quarters during Hajj can impair adequate infection control, so health agencies have recommend visiting pilgrims to carefully wash hands, consume hygienic foods and isolate themselves when ill [105, 106].

Awareness among health care providers should be raised as well. Superspreading is commonly observed in hospital settings because of the sustained contact in close quarters, medical treatments generating aerosolized virus and susceptibility of hospitalized patients [21]. Even in the endemic nation of Saudi Arabia, the Ministry of Health identified issues such as ambiguity on MERS case definition, inadequate infection control and overcrowding to be sources of hospital outbreaks [107]. Similar concerns have been raised in Korea as well; doctor shopping and suboptimal infection control due to crowded hospital rooms seem to have propagated the spread nationwide [19].

An interesting facet of this international analysis was the difference in outcomes for MERS-CoV infected patients depending on where they are located. About 41% of patients in the Middle Eastern and African countries died [1]. Fatality in Europe was 47% where about half of those infected died, most likely due to severity of illness in cases transported there for treatment. Asian countries, on the other hand, have an atypical 20.3% case fatality.

We attempted to estimate the basic reproduction number via Bayesian analysis but the mixing of human and camel sequences with multiple introductions of MERS-CoV from animals to humans was not possible to model. However, from the literature review, many researchers seem to have reached consistent conclusion that the R_0_ is less than 1 even in endemic countries; notable deviations arose only when MERS-CoV was introduced to a hospital setting then R_0_ increased by folds. Superspreading has been cited to be responsible for this trend since the exponential number of transmissions by a few can raise the R_0_ [76].

Although fundamental and gravely essential part of epidemiological investigation contact tracing is costly, labor intensive and prone to human error. Along with many cases where possible sources of transmissions are unknown, zoonotic transmissions were at times conjectural, based on patients’ occupation or recall of food consumption. Nevertheless, we were able to illustrate known and probable transmission events starting from first known cases of MERS to recent cases of 2016. In addition, we have conducted Bayesian phylogeographic analysis of MERS-CoV strains in both humans and camels, which is the most up-to-date and comprehensive, to our knowledge.

Purely epidemiological data such as incidence reports and contact tracing can be prone to inaccuracies but can provide background information on individual cases and population level transmission. Genetic data circumvents human errors and presents quantitative information about an infectious agent, but it is not fully informative without accompanying details on collection. Using phylodynamics to investigate evolutionary history of pathogen can add indispensable details to curb an outbreak such as identifying most closely related cases and predicting origin of the virus, revealing additional details at molecular level when epidemiological tracing is inadequate. As demonstrated with MERS, combining these two methods in a holistic approach is valuable for understanding pathogen history and transmission in order to implement effective public health measures.

## Conclusion

Through combination of epidemiological data and genetic analysis, we present evolutionary history of MERS-CoV affecting Middle East and beyond with focus on hospital outbreaks and zoonotic transmissions.

## Additional files

Additional file 1: **Table S1.** List of sequences used for analysis. Column “Label” corresponds to labels for sequences. presented in Figures 3 and 4 with country (by 2-letter ISO country code) and year of collection; countries, sources, and dates (month-year) are based on information in GenBank or related publication (indicated in Reference column). (DOCX 128 kb)

Additional file 2: **Figure S1.** Results of recombination analysis. Out of all 196 MERS-CoV ORF1a/b sequences, two strains were detected by Bootscan/Recscan method in RDP as recombinant strains. **Figure S2.** Likelihood mapping of MERS ORF1a/b (a) main dataset, (b) human subset, and (c) camel subset. Main dataset has both camel and human sequences. Each dot represents the likelihoods of the three possible unrooted trees for a set of four randomly selected sequences: dots close to the corners represent tree-like phylogenetic signal and those at the sides represent network-like signal. The central area of the likelihood map represents star-like signal of unresolved phylogenetic information. **Figure S3**. Temporal signal analysis using TempEst. Plots of the root-to-tip genetic distance against sampling time are shown for phylogenies estimated from three alignments: (a) main dataset with both human and camel sequences, (b) human sequences, and (c) camel sequences. **Figure S4.** Time-scaled phylogeographic tree of MERS-CoV ORF1a/b sequences isolated from humans and camels by country. Each color shown in legend represents country of sampled sequence (tip branches) as well as ancestral lineage (internal branches) inferred by Bayesian phylogeography. Brown camel symbols represent MERS-CoV sequences isolated from camels. * represents posterior probability for the clade >0.90. ** >0.95 and *** >0.99. **Figure S5.** Time-scaled phylogeographic tree of MERS-CoV ORF1a/b sequences isolated from humans by city. Each color shown in legend represents city or region of sampled sequence (tip branches) as well as ancestral lineage (internal branches) inferred by Bayesian phylogeography. * represents posterior probability for the clade >0.90. ** for >0.95 and *** for >0.99. Figure S6. Time-scaled phylogeographic tree of MERS-CoV ORF1a/b sequences isolated from humans by country. Each color shown in legend represents country of sampled sequence (tip branches) as well as ancestral lineage (internal branches) inferred by Bayesian phylogeography. * represents posterior probability for the clade >0.90. ** for >0.95 and *** for >0.99. (ZIP 945 kb)

## Abbreviations

CoV: Coronavirus
MCMC: Markov chain Monte Carlo
MERS: Middle East respiratory syndrome
MERS-CoV: Middle East respiratory syndrome-coronavirus
SARS: Severe acute respiratory syndrome
tMRCA: Time to most recent common ancestor
