# Supplementary material for: The global spread of Middle East respiratory syndrome: an analysis fusing traditional epidemiological tracing and molecular phylodynamics
Source: Glob Health Res Policy. 2016 Sep 28;1:14. doi: 10.1186/s41256-016-0014-7 (PMC5693564; doi:10.1186/s41256-016-0014-7)
Supplement: Supplementary file 1 — Table S1. List of sequences used for analysis. Column “Label” corresponds to labels for sequences. presented in Figures 3 and 4 with country (by 2-letter ISO country code) and year of collection; countries, sources, and dates (month-year) are based on information in GenBank or related publication (indicated in Reference column). (DOCX 128 kb) [file 41256_2016_14_MOESM1_ESM.docx]

**Supplementary Table 1**. List of sequences used for analysis. Column “Label” corresponds to labels for sequences presented in Figures 3 and 4 with country (by 2-letter ISO country code) and year of collection; countries, sources, and dates (month-year) are based on information in GenBank or related publication (indicated in Reference column).

| List of Sequences Used for Analysis | | | | | | |
| --- | --- | --- | --- | --- | --- | --- |
| Legend | **Source** | **City** | **Country** | **Collection Date** | **Accession Number** | **Reference** |
| 2KR15 | Human | Pyeongtaek | Republic of Korea | May-15 | KT029139 | [51] |
| 4FR13 | Human | Unknown | France | May-13 | KJ361503 | Direct submission by Institut Pasteur, France |
| 9GB13 | Human | Manchester | United Kingdom | Feb-13 | KM210277 | Direct submission by Public Health of England |
| 10GB13 | Human | Manchester | United Kingdom | Feb-13 | KM015348 | HPA UK Novel Coronavirus Investigation team |
| 11SA13 | Human | Jeddah | Saudi Arabia | Nov-13 | KF958702 | [81] |
| 12SA13 | Camel | Jeddah | Saudi Arabia | Nov-13 | KF917527 | [81] |
| 30SA13 | Human | Al-Hasa | Saudi Arabia | May-13 | KF600655 | [59] |
| 33SA12 | Human | Riyadh | Saudi Arabia | Oct-12 | KF600652 | [59] |
| 34SA13 | Human | Al-Hasa | Saudi Arabia | May-13 | KF600651 | [59] |
| 38SA13 | Human | Al-Hasa | Saudi Arabia | May-13 | KF600647 | [59] |
| 40SA13 | Human | Al-Hasa | Saudi Arabia | May-13 | KF600645 | [59] |
| 41SA13 | Human | Al-Hasa | Saudi Arabia | May-13 | KF600644 | [59] |
| 51SA13 | Human | Al-Hasa | Saudi Arabia | May-13 | KF600634 | [59] |
| 53SA13 | Human | Al-Hasa | Saudi Arabia | May-13 | KF600632 | [59] |
| 55SA13 | Human | Buraidah | Saudi Arabia | May-13 | KF600630 | [59] |
| 57SA13 | Human | Hafr-al-Batin | Saudi Arabia | Jun-13 | KF600628 | [59] |
| 58SA13 | Human | Al-Hasa | Saudi Arabia | May-13 | KF600627 | [59] |
| 65SA12 | Human | Bisha | Saudi Arabia | Jun-12 | KF600620 | [59] |
| 72SA13 | Human | Riyadh | Saudi Arabia | Feb-13 | KF600613 | [59] |
| 73SA12 | Human | Riyadh | Saudi Arabia | Oct-12 | KF600612 | [59] |
| 125SA13 | Human | Riyadh | Saudi Arabia | Aug-13 | KJ156946 | [83] |
| 126SA13 | Human | Al-Hasa | Saudi Arabia | Jun-13 | KJ156943 | [83] |
| 127SA13 | Human | Riyadh | Saudi Arabia | Jul-13 | KJ156937 | [83] |
| 128SA13 | Human | Riyadh | Saudi Arabia | Aug-13 | KJ156926 | [83] |
| 129SA13 | Human | Riyadh | Saudi Arabia | Aug-13 | KJ156918 | [83] |
| 130SA13 | Human | Taif | Saudi Arabia | Jun-13 | KJ156896 | [83] |
| 131SA13 | Human | Riyadh | Saudi Arabia | Aug-13 | KJ156891 | [83] |
| 132SA13 | Human | Riyadh | Saudi Arabia | Aug-13 | KJ156888 | [83] |
| 165SA13 | Human | Riyadh | Saudi Arabia | Mar-13 | KJ156952 | [83] |
| 166SA13 | Human | Taif | Saudi Arabia | Jun-13 | KJ156949 | [83] |
| 167SA13 | Human | Riyadh | Saudi Arabia | Jul-13 | KJ156944 | [83] |
| 168SA13 | Human | Riyadh | Saudi Arabia | Aug-13 | KJ156934 | [83] |
| 169SA13 | Human | Hafr-al-Batin | Saudi Arabia | Aug-13 | KJ156910 | [83] |
| 170SA13 | Human | Wadi-ad-Dawasir | Saudi Arabia | Jun-13 | KJ156881 | [83] |
| 171SA13 | Human | Hafr-al-Batin | Saudi Arabia | Aug-13 | KJ156874 | [83] |
| 172SA13 | Human | Riyadh | Saudi Arabia | Jul-13 | KJ156869 | [83] |
| 173SA13 | Human | Al-Hasa | Saudi Arabia | May-13 | KJ156866 | [83] |
| 239AE14 | Camel | Dubai | United Arab Emirates | Jun-14 | KP719932 | [94] |
| 240AE14 | Camel | Dubai | United Arab Emirates | Jun-14 | KP719931 | [94] |
| 241AE14 | Camel | Dubai | United Arab Emirates | Jun-14 | KP719930 | [94] |
| 242AE14 | Camel | Dubai | United Arab Emirates | Jun-14 | KP719929 | [94] |
| 243AE14 | Camel | Dubai | United Arab Emirates | Jun-14 | KP719928 | [94] |
| 244AE14 | Camel | Dubai | United Arab Emirates | Jun-14 | KP719927 | [94] |
| 245GB13 | Human | Manchester | United Kingdom | Feb-13 | KM210278 | HPA UK Novel Coronavirus Investigation team |
| 248SA14 | Human | Riyadh | Saudi Arabia | Apr-14 | KM027262 | [65] |
| 249SA14 | Human | Mecca | Saudi Arabia | Apr-14 | KM027261 | [65] |
| 250SA14 | Human | Jeddah | Saudi Arabia | Apr-14 | KM027260 | [65] |
| 251SA14 | Human | Jeddah | Saudi Arabia | Apr-14 | KM027259 | [65] |
| 252SA14 | Human | Jeddah | Saudi Arabia | Apr-14 | KM027258 | [65] |
| 253SA14 | Human | Jeddah | Saudi Arabia | Apr-14 | KM027257 | [65] |
| 254SA14 | Human | Jeddah | Saudi Arabia | Apr-14 | KM027256 | [65] |
| 255SA14 | Human | Jeddah | Saudi Arabia | Apr-14 | KM027255 | [65] |
| 286QA14 | Camel | Unknown | Qatar | Feb-14 | KJ650098 | [98] |
| 287SA13 | Camel | Al-Hasa | Saudi Arabia | Nov-13 | KJ650297 | [85] |
| 288SA13 | Camel | Al-Hasa | Saudi Arabia | Dec-13 | KJ650296 | [85] |
| 289SA13 | Camel | Al-Hasa | Saudi Arabia | Dec-13 | KJ650295 | [85] |
| 290DE13 | Human | Munich | Germany | Mar-13 | KF192507 | [48] |
| 291SA13 | Human | Al-Hasa | Saudi Arabia | Apr-13 | KF186565 | [58] |
| 292SA13 | Human | Al-Hasa | Saudi Arabia | May-13 | KF186567 | [58] |
| 293SA13 | Human | Al-Hasa | Saudi Arabia | Apr-13 | KF186566 | [58] |
| 294SA13 | Human | Al-Hasa | Saudi Arabia | May-13 | KF186564 | [58] |
| 303OM13 | Human | Unknown | Oman | Dec-13 | KT156561 | Direct submission by CDC |
| 304OM13 | Human | Unknown | Oman | Oct-13 | KT156560 | Direct submission by CDC |
| 311SA15 | Human | Riyadh | Saudi Arabia | Mar-15 | KT026454 | Direct submission by CDC |
| 312SA15 | Human | Riyadh | Saudi Arabia | Feb-15 | KT026453 | Direct submission by CDC |
| 313CN15 | Human | Guangdong | China | May-15 | KT006149 | [69] |
| 314SA15 | Human | Riyadh | Saudi Arabia | Jan-15 | KR011266 | Direct submission by CDC |
| 315SA15 | Human | Riyadh | Saudi Arabia | Jan-15 | KR011265 | Direct submission by CDC |
| 316SA15 | Human | Riyadh | Saudi Arabia | Jan-15 | KR011264 | Direct submission by CDC |
| 317SA15 | Human | Riyadh | Saudi Arabia | Jan-15 | KR011263 | Direct submission by CDC |
| 322AE14 | Human | Abu Dhabi | United Arab Emirates | Apr-14 | KP209313 | Direct submission by CDC |
| 323AE13 | Human | Abu Dhabi | United Arab Emirates | Nov-13 | KP209312 | Direct submission by CDC |
| 324AE14 | Human | Abu Dhabi | United Arab Emirates | Apr-14 | KP209311 | Direct submission by CDC |
| 325AE14 | Human | Abu Dhabi/ Gayathi | United Arab Emirates | Mar-14 | KP209310 | Direct submission by CDC |
| 326AE14 | Human | Abu Dhabi | United Arab Emirates | Apr-14 | KP209309 | Direct submission by CDC |
| 327AE14 | Human | Abu Dhabi | United Arab Emirates | Apr-14 | KP209308 | Direct submission by CDC |
| 328AE14 | Human | Abu Dhabi | United Arab Emirates | Apr-14 | KP209307 | Direct submission by CDC |
| 329AE14 | Human | Abu Dhabi | United Arab Emirates | Apr-14 | KP209306 | Direct submission by CDC |
| 348US14 | Human | Orlando, FL | USA | May-14 | KJ829365 | Direct submission by CDC |
| 349US14 | Human | Munster, IN | USA | Apr-14 | KJ813439 | [84] |
| 352SA13 | Camel | Taif | Saudi Arabia | Nov-13 | KJ713299 | [92] |
| 353SA13 | Camel | Taif | Saudi Arabia | Nov-13 | KJ713298 | [92] |
| 354SA13 | Camel | Taif | Saudi Arabia | Nov-13 | KJ713297 | [92] |
| 355SA13 | Camel | Taif | Saudi Arabia | Nov-13 | KJ713296 | [92] |
| 356SA13 | Camel | Taif | Saudi Arabia | Nov-13 | KJ713295 | [92] |
| 373QA13 | Human | Unknown | Qatar | Oct-13 | KF961222 | [80] |
| 374QA13 | Human | Unknown | Qatar | Oct-13 | KF961221 | [80] |
| 383GB12 | Human | London | United Kingdom | Sep-12 | KC164505 | Direct submission by Public Health of England |
| 385SA12 | Human | Jeddah | Saudi Arabia | Jun-12 | JX869059 | [53] |
| 387TH15 | Human | Bangkok | Thailand | Jun-15 | KT225476 | Direct submission by Thailand Ministry of Public Health |
| 388EG13 | Camel | Cairo | Egypt | Dec-13 | KJ477102 | [93] |
| 389JO12 | Human | Zarqa | Jordan | Apr-12 | KC776174 | Direct submission by US Naval Medical Research |
| 390SA14 | Human | Riyadh | Saudi Arabia | May-14 | KT121576 | [62] |
| 391SA14 | Human | Riyadh | Saudi Arabia | May-14 | KT121577 | [62] |
| 392SA14 | Human | Riyadh | Saudi Arabia | May-14 | KT121578 | [62] |
| 393SA14 | Human | Riyadh | Saudi Arabia | Apr-14 | KT121579 | [62] |
| 394SA14 | Human | Riyadh | Saudi Arabia | Apr-14 | KT121580 | [62] |
| 395SA14 | Human | Riyadh | Saudi Arabia | May-14 | KT121581 | [62] |
| 396SA14 | Camel | Jeddah | Saudi Arabia | May-14 | KT368824 | [49] |
| 397SA14 | Camel | Riyadh | Saudi Arabia | Jul-14 | KT368825 | [49] |
| 398SA14 | Camel | Riyadh | Saudi Arabia | Jul-14 | KT368826 | [49] |
| 399SA14 | Camel | Jeddah | Saudi Arabia | Sep-14 | KT368827 | [49] |
| 400SA14 | Camel | Jeddah | Saudi Arabia | Dec-14 | KT368828 | [49] |
| 401SA14 | Camel | Jeddah | Saudi Arabia | Dec-14 | KT368829 | [49] |
| 402SA14 | Camel | Jeddah | Saudi Arabia | Dec-14 | KT368830 | [49] |
| 403SA14 | Camel | Jeddah | Saudi Arabia | Dec-14 | KT368831 | [49] |
| 404SA14 | Camel | Jeddah | Saudi Arabia | Dec-14 | KT368832 | [49] |
| 405SA14 | Camel | Jeddah | Saudi Arabia | Dec-14 | KT368833 | [49] |
| 406SA14 | Camel | Jeddah | Saudi Arabia | Dec-14 | KT368834 | [49] |
| 407SA14 | Camel | Jeddah | Saudi Arabia | Dec-14 | KT368835 | [49] |
| 408SA14 | Camel | Jeddah | Saudi Arabia | Dec-14 | KT368836 | [49] |
| 409SA14 | Camel | Jeddah | Saudi Arabia | Dec-14 | KT368837 | [49] |
| 410SA14 | Camel | Jeddah | Saudi Arabia | Dec-14 | KT368838 | [49] |
| 411SA14 | Camel | Jeddah | Saudi Arabia | Dec-14 | KT368839 | [49] |
| 412SA14 | Camel | Jeddah | Saudi Arabia | Dec-14 | KT368840 | [49] |
| 413SA14 | Camel | Jeddah | Saudi Arabia | Dec-14 | KT368841 | [49] |
| 414SA14 | Camel | Jeddah | Saudi Arabia | Dec-14 | KT368842 | [49] |
| 415SA14 | Camel | Jeddah | Saudi Arabia | Dec-14 | KT368843 | [49] |
| 416SA14 | Camel | Jeddah | Saudi Arabia | Dec-14 | KT368844 | [49] |
| 417SA14 | Camel | Jeddah | Saudi Arabia | Dec-14 | KT368845 | [49] |
| 418SA14 | Camel | Jeddah | Saudi Arabia | Nov-14 | KT368846 | [49] |
| 419SA14 | Camel | Jeddah | Saudi Arabia | Nov-14 | KT368847 | [49] |
| 420SA14 | Camel | Jeddah | Saudi Arabia | Nov-14 | KT368848 | [49] |
| 421SA14 | Camel | Jeddah | Saudi Arabia | Oct-14 | KT368849 | [49] |
| 422SA14 | Camel | Jeddah | Saudi Arabia | Oct-14 | KT368850 | [49] |
| 423SA14 | Camel | Jeddah | Saudi Arabia | Oct-14 | KT368851 | [49] |
| 424SA14 | Camel | Jeddah | Saudi Arabia | Oct-14 | KT368852 | [49] |
| 425SA14 | Camel | Jeddah | Saudi Arabia | Sep-14 | KT368853 | [49] |
| 426SA14 | Camel | Jeddah | Saudi Arabia | Sep-14 | KT368854 | [49] |
| 427SA14 | Camel | Jeddah | Saudi Arabia | Sep-14 | KT368855 | [49] |
| 428SA14 | Camel | Jeddah | Saudi Arabia | Sep-14 | KT368856 | [49] |
| 429SA14 | Camel | Jeddah | Saudi Arabia | Sep-14 | KT368857 | [49] |
| 430SA15 | Camel | Jeddah | Saudi Arabia | Jan-15 | KT368858 | [49] |
| 431SA15 | Camel | Jeddah | Saudi Arabia | Jan-15 | KT368859 | [49] |
| 432SA15 | Camel | Jeddah | Saudi Arabia | Jan-15 | KT368860 | [49] |
| 433SA15 | Camel | Jeddah | Saudi Arabia | Jan-15 | KT368861 | [49] |
| 434SA15 | Camel | Jeddah | Saudi Arabia | Jan-15 | KT368862 | [49] |
| 435SA15 | Camel | Jeddah | Saudi Arabia | Jan-15 | KT368863 | [49] |
| 436SA15 | Camel | Jeddah | Saudi Arabia | Jan-15 | KT368864 | [49] |
| 437SA15 | Camel | Jeddah | Saudi Arabia | Jan-15 | KT368865 | [49] |
| 438SA15 | Camel | Jeddah | Saudi Arabia | Feb-15 | KT368866 | [49] |
| 439SA15 | Camel | Jeddah | Saudi Arabia | Feb-15 | KT368867 | [49] |
| 440SA15 | Camel | Riyadh | Saudi Arabia | Mar-15 | KT368868 | [49] |
| 441SA15 | Camel | Riyadh | Saudi Arabia | Mar-15 | KT368869 | [49] |
| 442SA15 | Camel | Riyadh | Saudi Arabia | Mar-15 | KT368870 | [49] |
| 443SA15 | Camel | Riyadh | Saudi Arabia | Mar-15 | KT368871 | [49] |
| 444SA15 | Camel | Riyadh | Saudi Arabia | Mar-15 | KT368872 | [49] |
| 445SA15 | Camel | Riyadh | Saudi Arabia | Mar-15 | KT368873 | [49] |
| 446SA15 | Camel | Riyadh | Saudi Arabia | Mar-15 | KT368874 | [49] |
| 447SA15 | Camel | Riyadh | Saudi Arabia | Mar-15 | KT368875 | [49] |
| 448SA15 | Camel | Riyadh | Saudi Arabia | Mar-15 | KT368876 | [49] |
| 449SA15 | Camel | Riyadh | Saudi Arabia | Mar-15 | KT368877 | [49] |
| 450SA15 | Camel | Riyadh | Saudi Arabia | Mar-15 | KT368878 | [49] |
| 451SA15 | Camel | Riyadh | Saudi Arabia | Mar-15 | KT368879 | [49] |
| 452SA15 | Camel | Taif | Saudi Arabia | Apr-15 | KT368880 | [49] |
| 453SA15 | Camel | Taif | Saudi Arabia | Apr-15 | KT368881 | [49] |
| 454SA15 | Camel | Taif | Saudi Arabia | Apr-15 | KT368882 | [49] |
| 455SA15 | Camel | Taif | Saudi Arabia | Apr-15 | KT368883 | [49] |
| 456SA15 | Camel | Taif | Saudi Arabia | Apr-15 | KT368884 | [49] |
| 457SA15 | Camel | Taif | Saudi Arabia | Apr-15 | KT368885 | [49] |
| 458SA15 | Camel | Taif | Saudi Arabia | Apr-15 | KT368886 | [49] |
| 459SA15 | Camel | Riyadh | Saudi Arabia | Apr-15 | KT368887 | [49] |
| 460SA15 | Camel | Taif | Saudi Arabia | Apr-15 | KT368888 | [49] |
| 461SA15 | Camel | Taif | Saudi Arabia | Mar-15 | KT368889 | [49] |
| 462SA15 | Camel | Taif | Saudi Arabia | Mar-15 | KT368890 | [49] |
| 463SA15 | Human | Jeddah | Saudi Arabia | Feb-15 | KT806044 | Direct submission by CDC |
| 464SA15 | Human | Jeddah | Saudi Arabia | Feb-15 | KT806045 | Direct submission by CDC |
| 465SA15 | Human | Hofuf | Saudi Arabia | May-15 | KT806046 | Direct submission by CDC |
| 466SA15 | Human | Hofuf | Saudi Arabia | Mar-15 | KT806047 | Direct submission by CDC |
| 467SA15 | Human | Khobar | Saudi Arabia | Feb-15 | KT806048 | Direct submission by CDC |
| 468SA15 | Human | Riyadh | Saudi Arabia | Feb-15 | KT806049 | Direct submission by CDC |
| 469SA15 | Human | Riyadh | Saudi Arabia | Feb-15 | KT806050 | Direct submission by CDC |
| 470SA15 | Human | Riyadh | Saudi Arabia | Feb-15 | KT806051 | Direct submission by CDC |
| 471SA15 | Human | Kharj | Saudi Arabia | Feb-15 | KT806052 | Direct submission by CDC |
| 472SA15 | Human | Kharj | Saudi Arabia | Feb-15 | KT806053 | Direct submission by CDC |
| 473SA15 | Human | Najran | Saudi Arabia | Feb-15 | KT806054 | Direct submission by CDC |
| 474SA15 | Human | Jeddah | Saudi Arabia | Feb-15 | KT806055 | Direct submission by CDC |
| 475KR15 | Human | Asan | Republic of Korea | Jun-15 | KT374051 | [50] |
| 476KR15 | Human | Pyeongtaek | Republic of Korea | May-15 | KT374052 | [50] |
| 477KR15 | Human | Seoul | Republic of Korea | Jun-15 | KT374054 | [50] |
| 478KR15 | Human | Seoul | Republic of Korea | Jun-15 | KT374056 | [50] |
| 479AE14 | Camel | Dubai | United Arab Emirates | Jun-14 | KP719933 | Direct submission by University of Bonn, Germany |
| 480AE14 | Camel | Dubai | United Arab Emirates | Mar-14 | KT751244 | [96] |
| 481AE14 | Camel | Dubai | United Arab Emirates | Mar-14 | KU242423 | [94] |
| 482AE14 | Camel | Dubai | United Arab Emirates | Mar-14 | KU242424 | [94] |
| 493SA13 | Human | Jeddah | Saudi Arabia | Nov-13 | KJ556336 | [95] |
| 494SA15 | Human | Riyadh | Saudi Arabia | Feb-15 | KT026455 | Direct submission by CDC |
| 495SA15 | Human | Riyadh | Saudi Arabia | Mar-15 | KT026456 | Direct submission by CDC |
| 496SA14 | Human | Riyadh | Saudi Arabia | May-14 | KT121572 | [62] |
| 497SA14 | Human | Riyadh | Saudi Arabia | May-14 | KT121573 | [62] |
| 498SA14 | Human | Riyadh | Saudi Arabia | May-14 | KT121574 | [62] |
| 499SA14 | Human | Riyadh | Saudi Arabia | May-14 | KT121575 | [62] |
