# Supplementary figures and images for: The global spread of Middle East respiratory syndrome: an analysis fusing traditional epidemiological tracing and molecular phylodynamics
Source: Glob Health Res Policy. 2016 Sep 28;1:14. doi: 10.1186/s41256-016-0014-7 (PMC5693564; doi:10.1186/s41256-016-0014-7)

## Slide 1
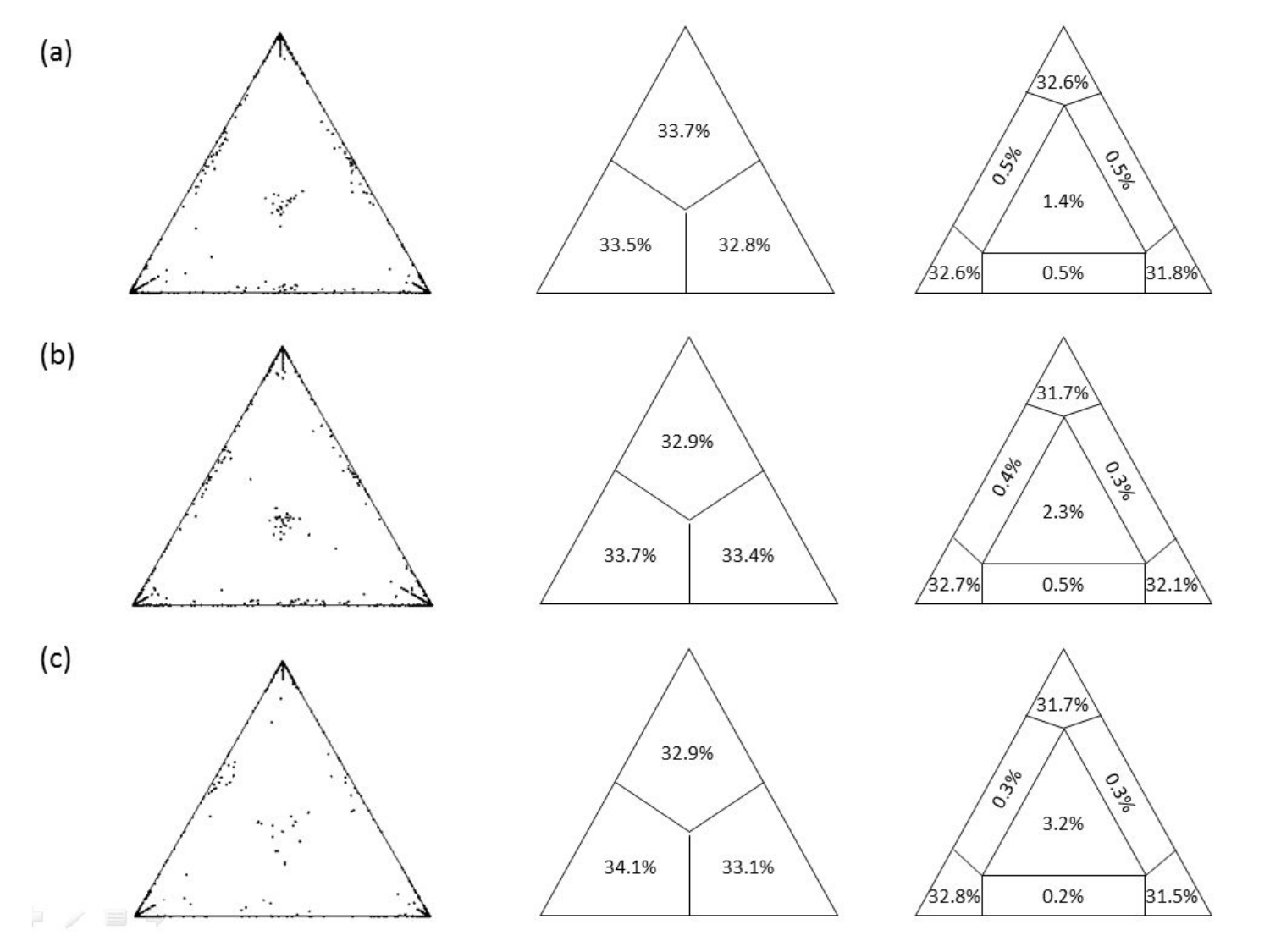

Supplement: Supplementary file 2 — Figure S1. Results of recombination analysis. Out of all 196 MERS-CoV ORF1a/b sequences, two strains were detected by Bootscan/Recscan method in RDP as recombinant strains. Figure S2. Likelihood mapping of MERS ORF1a/b (a) main dataset, (b) human subset, and (c) camel subset. Main dataset has both camel and human sequences. Each dot represents the likelihoods of the three possible unrooted trees for a set of four randomly selected sequences: dots close to the corners represent tree-like phylogenetic signal and those at the sides represent network-like signal. The central area of the likelihood map represents star-like signal of unresolved phylogenetic information. Figure S3. Temporal signal analysis using TempEst. Plots of the root-to-tip genetic distance against sampling time are shown for phylogenies estimated from three alignments: (a) main dataset with both human and camel sequences, (b) human sequences, and (c) camel sequences. Figure S4. Time-scaled phylogeographic tree of MERS-CoV ORF1a/b sequences isolated from humans and camels by country. Each color shown in legend represents country of sampled sequence (tip branches) as well as ancestral lineage (internal branches) inferred by Bayesian phylogeography. Brown camel symbols represent MERS-CoV sequences isolated from camels. * represents posterior probability for the clade >0.90. ** >0.95 and *** >0.99. Figure S5. Time-scaled phylogeographic tree of MERS-CoV ORF1a/b sequences isolated from humans by city. Each color shown in legend represents city or region of sampled sequence (tip branches) as well as ancestral lineage (internal branches) inferred by Bayesian phylogeography. * represents posterior probability for the clade >0.90. ** for >0.95 and *** for >0.99. Figure S6. Time-scaled phylogeographic tree of MERS-CoV ORF1a/b sequences isolated from humans by country. Each color shown in legend represents country of sampled sequence (tip branches) as well as ancestral lineage (internal branches) inferred by Ba [file 41256_2016_14_MOESM2_ESM.zip › SuppFigure2.pptx]

## Slide 1
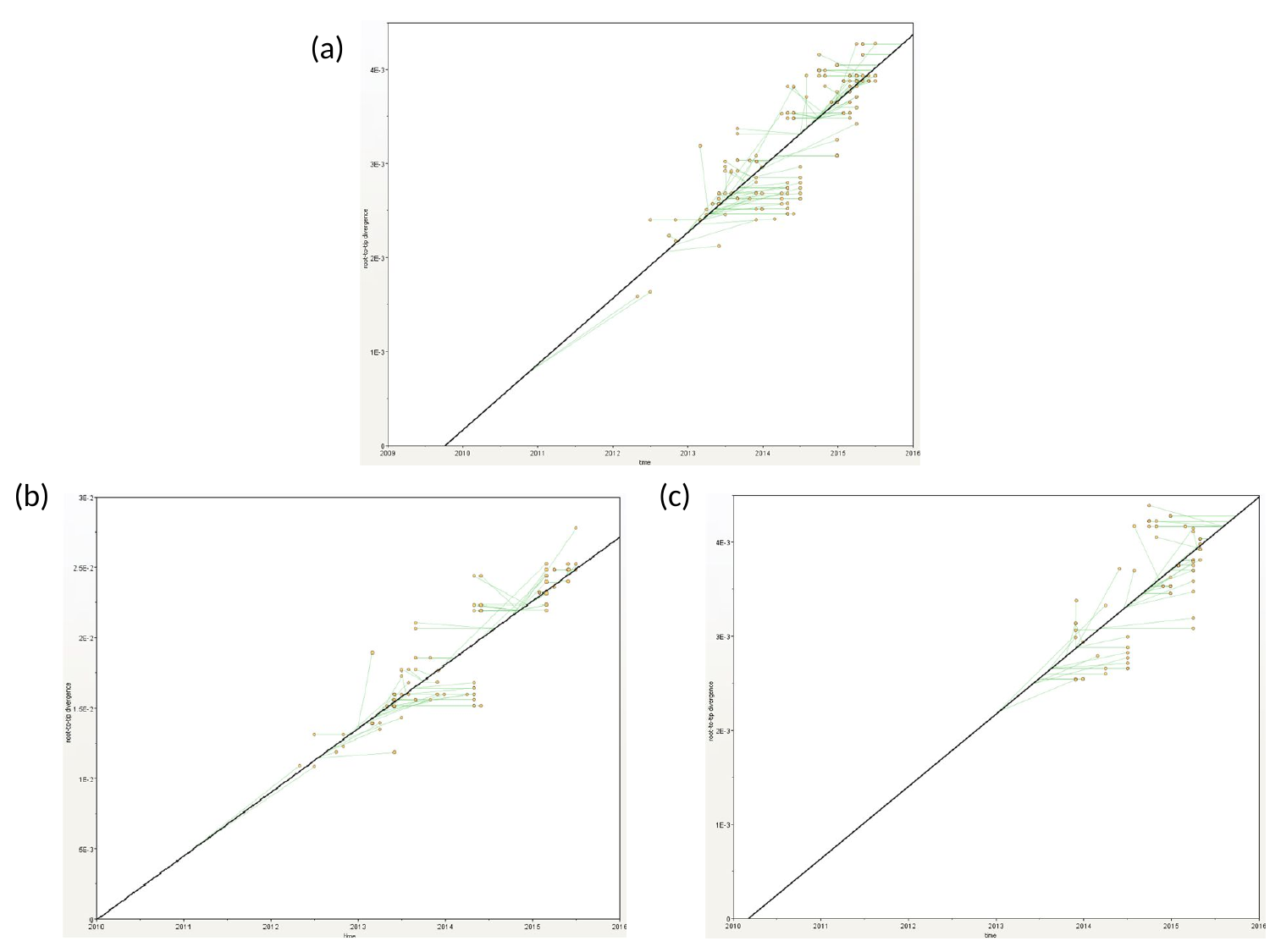

(a)
(b)
(c)

Supplement: Supplementary file 2 — Figure S1. Results of recombination analysis. Out of all 196 MERS-CoV ORF1a/b sequences, two strains were detected by Bootscan/Recscan method in RDP as recombinant strains. Figure S2. Likelihood mapping of MERS ORF1a/b (a) main dataset, (b) human subset, and (c) camel subset. Main dataset has both camel and human sequences. Each dot represents the likelihoods of the three possible unrooted trees for a set of four randomly selected sequences: dots close to the corners represent tree-like phylogenetic signal and those at the sides represent network-like signal. The central area of the likelihood map represents star-like signal of unresolved phylogenetic information. Figure S3. Temporal signal analysis using TempEst. Plots of the root-to-tip genetic distance against sampling time are shown for phylogenies estimated from three alignments: (a) main dataset with both human and camel sequences, (b) human sequences, and (c) camel sequences. Figure S4. Time-scaled phylogeographic tree of MERS-CoV ORF1a/b sequences isolated from humans and camels by country. Each color shown in legend represents country of sampled sequence (tip branches) as well as ancestral lineage (internal branches) inferred by Bayesian phylogeography. Brown camel symbols represent MERS-CoV sequences isolated from camels. * represents posterior probability for the clade >0.90. ** >0.95 and *** >0.99. Figure S5. Time-scaled phylogeographic tree of MERS-CoV ORF1a/b sequences isolated from humans by city. Each color shown in legend represents city or region of sampled sequence (tip branches) as well as ancestral lineage (internal branches) inferred by Bayesian phylogeography. * represents posterior probability for the clade >0.90. ** for >0.95 and *** for >0.99. Figure S6. Time-scaled phylogeographic tree of MERS-CoV ORF1a/b sequences isolated from humans by country. Each color shown in legend represents country of sampled sequence (tip branches) as well as ancestral lineage (internal branches) inferred by Ba [file 41256_2016_14_MOESM2_ESM.zip › SuppFigure3.pptx]

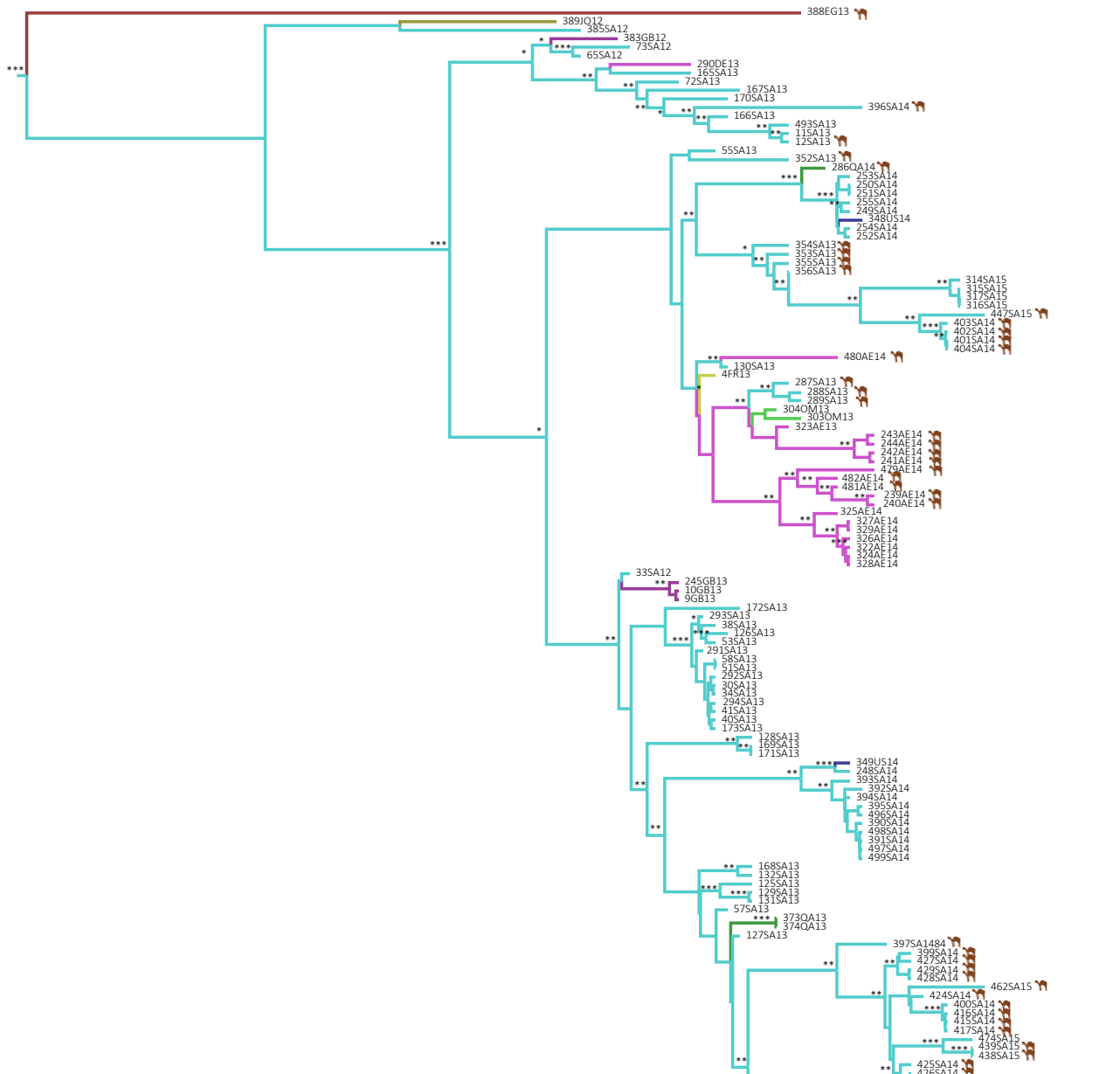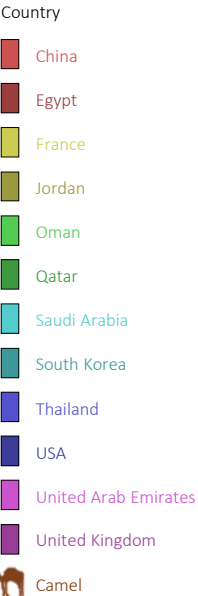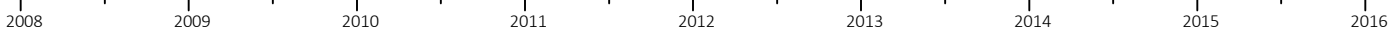

Supplement: Supplementary file 2 — Figure S1. Results of recombination analysis. Out of all 196 MERS-CoV ORF1a/b sequences, two strains were detected by Bootscan/Recscan method in RDP as recombinant strains. Figure S2. Likelihood mapping of MERS ORF1a/b (a) main dataset, (b) human subset, and (c) camel subset. Main dataset has both camel and human sequences. Each dot represents the likelihoods of the three possible unrooted trees for a set of four randomly selected sequences: dots close to the corners represent tree-like phylogenetic signal and those at the sides represent network-like signal. The central area of the likelihood map represents star-like signal of unresolved phylogenetic information. Figure S3. Temporal signal analysis using TempEst. Plots of the root-to-tip genetic distance against sampling time are shown for phylogenies estimated from three alignments: (a) main dataset with both human and camel sequences, (b) human sequences, and (c) camel sequences. Figure S4. Time-scaled phylogeographic tree of MERS-CoV ORF1a/b sequences isolated from humans and camels by country. Each color shown in legend represents country of sampled sequence (tip branches) as well as ancestral lineage (internal branches) inferred by Bayesian phylogeography. Brown camel symbols represent MERS-CoV sequences isolated from camels. * represents posterior probability for the clade >0.90. ** >0.95 and *** >0.99. Figure S5. Time-scaled phylogeographic tree of MERS-CoV ORF1a/b sequences isolated from humans by city. Each color shown in legend represents city or region of sampled sequence (tip branches) as well as ancestral lineage (internal branches) inferred by Bayesian phylogeography. * represents posterior probability for the clade >0.90. ** for >0.95 and *** for >0.99. Figure S6. Time-scaled phylogeographic tree of MERS-CoV ORF1a/b sequences isolated from humans by country. Each color shown in legend represents country of sampled sequence (tip branches) as well as ancestral lineage (internal branches) inferred by Ba [file 41256_2016_14_MOESM2_ESM.zip › SuppFigure4.pdf]

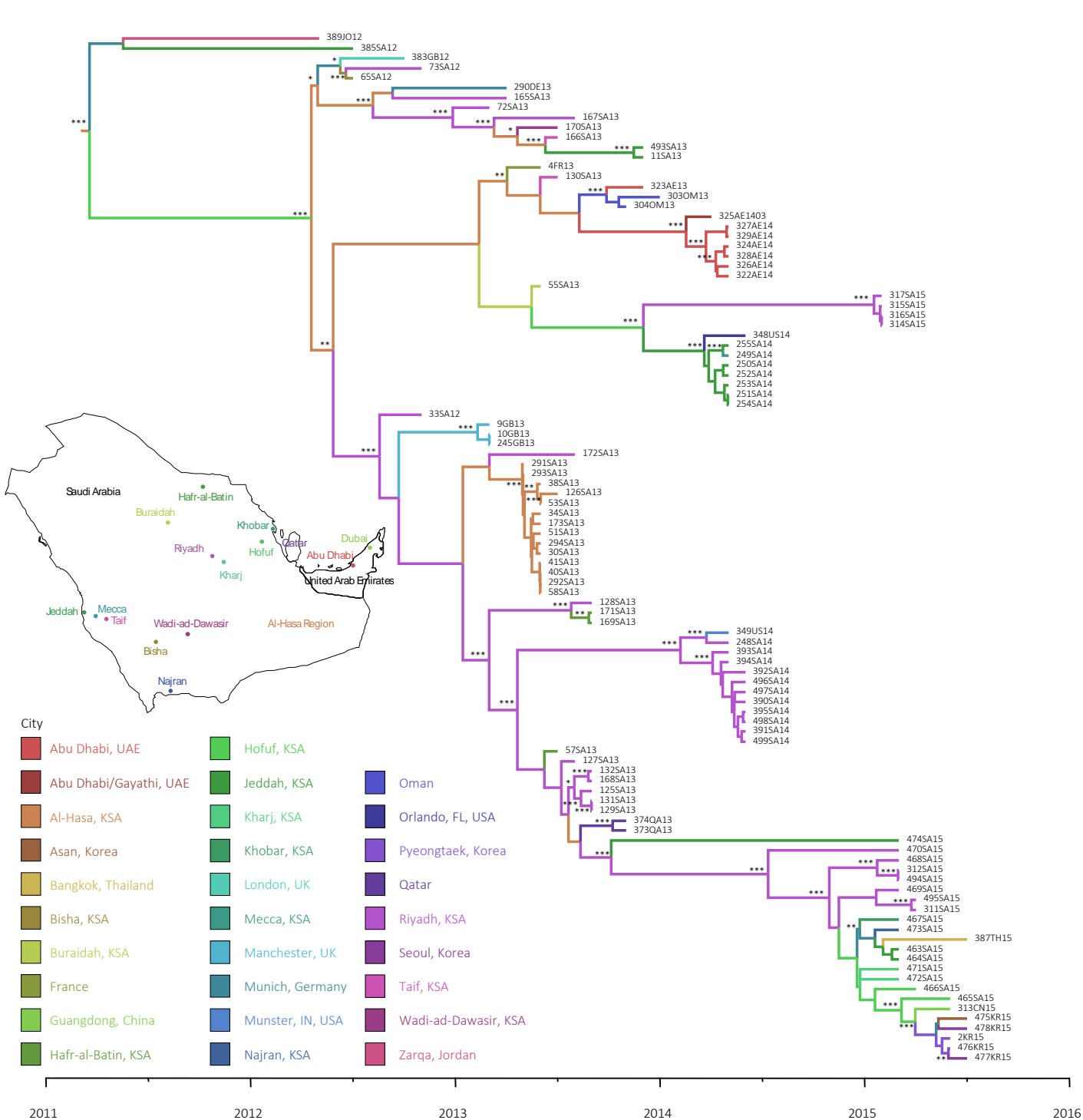

Supplement: Supplementary file 2 — Figure S1. Results of recombination analysis. Out of all 196 MERS-CoV ORF1a/b sequences, two strains were detected by Bootscan/Recscan method in RDP as recombinant strains. Figure S2. Likelihood mapping of MERS ORF1a/b (a) main dataset, (b) human subset, and (c) camel subset. Main dataset has both camel and human sequences. Each dot represents the likelihoods of the three possible unrooted trees for a set of four randomly selected sequences: dots close to the corners represent tree-like phylogenetic signal and those at the sides represent network-like signal. The central area of the likelihood map represents star-like signal of unresolved phylogenetic information. Figure S3. Temporal signal analysis using TempEst. Plots of the root-to-tip genetic distance against sampling time are shown for phylogenies estimated from three alignments: (a) main dataset with both human and camel sequences, (b) human sequences, and (c) camel sequences. Figure S4. Time-scaled phylogeographic tree of MERS-CoV ORF1a/b sequences isolated from humans and camels by country. Each color shown in legend represents country of sampled sequence (tip branches) as well as ancestral lineage (internal branches) inferred by Bayesian phylogeography. Brown camel symbols represent MERS-CoV sequences isolated from camels. * represents posterior probability for the clade >0.90. ** >0.95 and *** >0.99. Figure S5. Time-scaled phylogeographic tree of MERS-CoV ORF1a/b sequences isolated from humans by city. Each color shown in legend represents city or region of sampled sequence (tip branches) as well as ancestral lineage (internal branches) inferred by Bayesian phylogeography. * represents posterior probability for the clade >0.90. ** for >0.95 and *** for >0.99. Figure S6. Time-scaled phylogeographic tree of MERS-CoV ORF1a/b sequences isolated from humans by country. Each color shown in legend represents country of sampled sequence (tip branches) as well as ancestral lineage (internal branches) inferred by Ba [file 41256_2016_14_MOESM2_ESM.zip › SuppFigure5.pdf]

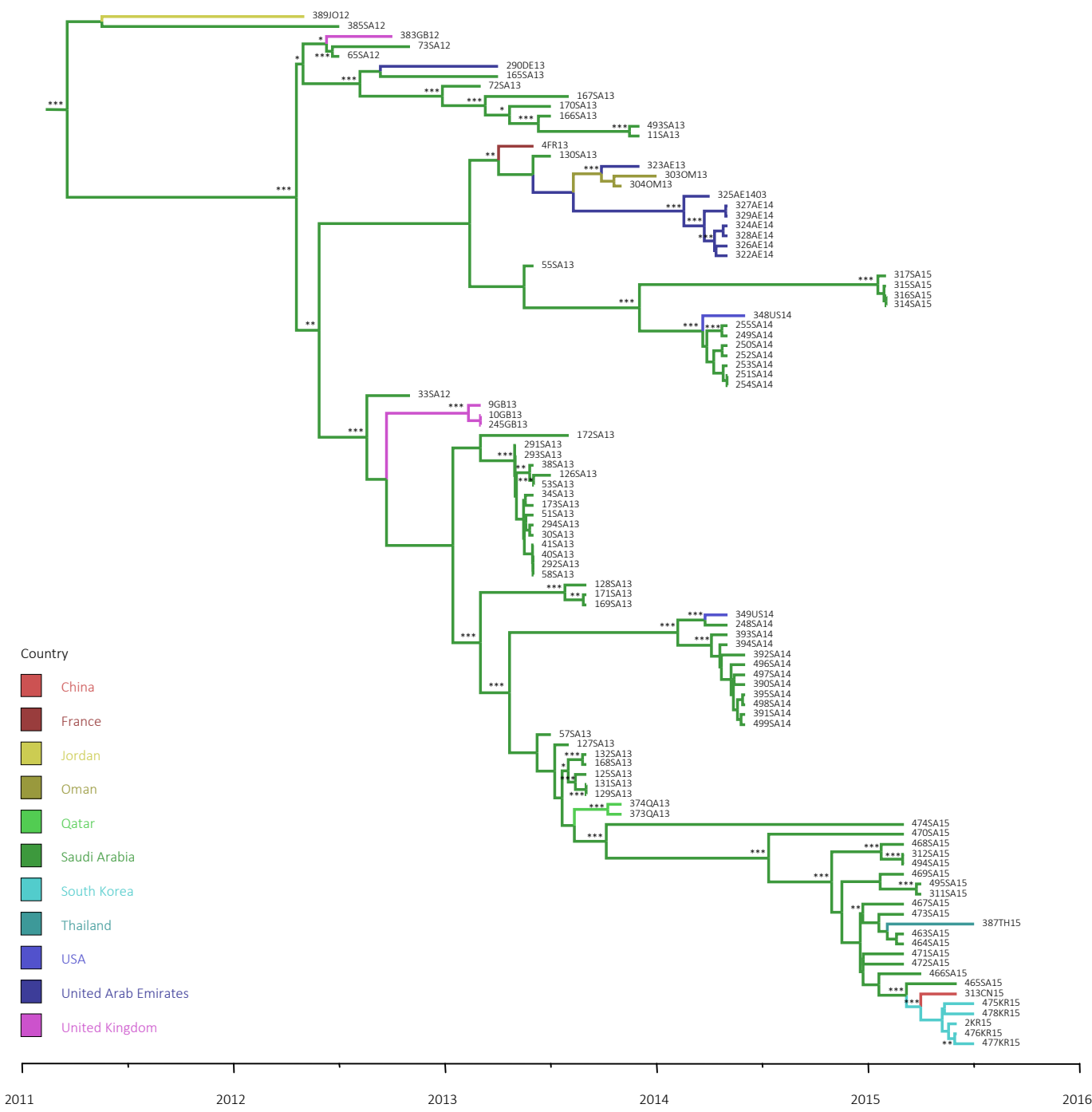

Supplement: Supplementary file 2 — Figure S1. Results of recombination analysis. Out of all 196 MERS-CoV ORF1a/b sequences, two strains were detected by Bootscan/Recscan method in RDP as recombinant strains. Figure S2. Likelihood mapping of MERS ORF1a/b (a) main dataset, (b) human subset, and (c) camel subset. Main dataset has both camel and human sequences. Each dot represents the likelihoods of the three possible unrooted trees for a set of four randomly selected sequences: dots close to the corners represent tree-like phylogenetic signal and those at the sides represent network-like signal. The central area of the likelihood map represents star-like signal of unresolved phylogenetic information. Figure S3. Temporal signal analysis using TempEst. Plots of the root-to-tip genetic distance against sampling time are shown for phylogenies estimated from three alignments: (a) main dataset with both human and camel sequences, (b) human sequences, and (c) camel sequences. Figure S4. Time-scaled phylogeographic tree of MERS-CoV ORF1a/b sequences isolated from humans and camels by country. Each color shown in legend represents country of sampled sequence (tip branches) as well as ancestral lineage (internal branches) inferred by Bayesian phylogeography. Brown camel symbols represent MERS-CoV sequences isolated from camels. * represents posterior probability for the clade >0.90. ** >0.95 and *** >0.99. Figure S5. Time-scaled phylogeographic tree of MERS-CoV ORF1a/b sequences isolated from humans by city. Each color shown in legend represents city or region of sampled sequence (tip branches) as well as ancestral lineage (internal branches) inferred by Bayesian phylogeography. * represents posterior probability for the clade >0.90. ** for >0.95 and *** for >0.99. Figure S6. Time-scaled phylogeographic tree of MERS-CoV ORF1a/b sequences isolated from humans by country. Each color shown in legend represents country of sampled sequence (tip branches) as well as ancestral lineage (internal branches) inferred by Ba [file 41256_2016_14_MOESM2_ESM.zip › SuppFigure6.pdf]
